# Revisiting Epithelial-to-Mesenchymal Transition in Liver Fibrosis: Clues for a Better Understanding of the “Reactive” Biliary Epithelial Phenotype

**DOI:** 10.1155/2016/2953727

**Published:** 2016-01-06

**Authors:** Luca Fabris, Simone Brivio, Massimiliano Cadamuro, Mario Strazzabosco

**Affiliations:** ^1^Department of Molecular Medicine, University of Padua School of Medicine, Viale G. Colombo 3, 35131 Padua, Italy; ^2^Liver Center, Section of Digestive Diseases, Yale University, TAC Building, 333 Cedar Street, New Haven, CT 06520, USA; ^3^School of Medicine and Surgery, University of Milan-Bicocca, Via Cadore 48, 20900 Monza, Italy

## Abstract

Whether liver epithelial cells contribute to the development of hepatic scarring by undergoing epithelial-to-mesenchymal transition (EMT) is a controversial issue. Herein, we revisit the concept of EMT in cholangiopathies, a group of severe hepatic disorders primarily targeting the bile duct epithelial cell (cholangiocyte), leading to progressive portal fibrosis, the main determinant of liver disease progression. Unfortunately, therapies able to halt this process are currently lacking. In cholangiopathies, fibrogenesis is part of ductular reaction, a reparative complex involving epithelial, mesenchymal, and inflammatory cells. Ductular reactive cells (DRC) are cholangiocytes derived from the activation of the hepatic progenitor cell compartment. These cells are arranged into irregular strings and express a “reactive” phenotype, which enables them to extensively crosstalk with the other components of ductular reaction. We will first discuss EMT in liver morphogenesis and then highlight how some of these developmental programs are partly reactivated in DRC. Evidence for “bona fide” EMT changes in cholangiocytes is lacking, but expression of some mesenchymal markers represents a fundamental repair mechanism in response to chronic biliary damage with potential harmful fibrogenetic effects. Understanding microenvironmental cues and signaling perturbations promoting these changes in DRC may help to identify potential targets for new antifibrotic therapies in cholangiopathies.

## 1. Introduction

Epithelial-to-mesenchymal transition (EMT) is a process of cellular reprogramming through which differentiated epithelial cells lose their native identity and acquire morphological and functional properties of mesenchymal cells, including a spindle-shaped (“fibroblast-like”) appearance and the ability to detach from and to migrate outside the epithelial layer [1]. This process is relevant in physiological conditions, as seen during embryonic development, but it may occur also in pathological conditions, leading to organ fibrosis and malignant transformation in several organs [2].

The loss of epithelial cell-cell adhesion, caused by the relocalization and/or degradation of critical junction proteins, including E-cadherin, *β*-catenin, zonula occludens-1, occludin, and claudin, usually represents the first step of EMT. E-cadherin loss is often counterbalanced by the aberrant* de novo* expression of N-cadherin, an adhesion molecule enabling epithelial cells to establish dynamic interactions with surrounding mesenchymal cells. The disassembly of cell junctions, together with the ability to erode the basement membrane, results in a reduced intercellular cohesion within the epithelial layer [3, 4]. However, cells undergoing EMT also show a rearrangement of the cytoskeletal architecture, deriving from the downregulation of cytokeratins (K) along with the upregulation of cytoskeletal proteins belonging to the mesenchymal lineage, including vimentin, S100A4 (also called fibroblast specific protein-1), and, eventually, *α*-smooth muscle actin (*α*-SMA). These cytoskeletal and cell surface remodeling lead to the loss of the apical-basal polarity, typical of the epithelial phenotype, in favor of a front-rear polarity, prerequisite for the increased motility displayed by mesenchymal cells. Additional EMT changes include the ability to produce extracellular matrix (ECM) components, such as fibrillar collagen, fibronectin, elastin, and tenascin, in conjunction with a range of matrix metalloproteinases (MMPs), particularly MMP2 and MMP9, and to increase the expression of integrin receptors mediating the interactions with ECM [1, 3, 5]. However, it must be underlined that the transition from an epithelial to a mesenchymal cell phenotype is not merely an “on/off” state but rather a highly dynamic process evolving gradually [6].

## 2. Molecular Players and Intracellular Pathways Regulating the “EMT Machinery”

Gene expression changes in EMT are orchestrated by a number of transcription factors actively engaged in embryogenesis, such as Snail (Snail1), Slug (Snail2), Twist1/2, and ZEB1/2. Their activation in response to growth factors, cytokines, and morphogens [13, 14] is an early event during EMT. Indeed, EMT can be induced by a number of extracellular signals, whose downstream transduction pathways extensively crosstalk with each other, share effector molecules, and converge on common endpoints [15].

Members of the transforming growth factor- (TGF-) *β* family (in particular TGF-*β*1) are the prototypical activators of EMT. TGF-*β*1 binding to the TGF-*β* type II receptor results in the activation of the Smad signaling pathway, which induces the expression of EMT transcription factors (especially Snail and ZEB family members), and cooperates with them in promoting gene reprogramming. However, TGF-*β*1 can also act through Smad-independent intracellular pathways, by activating Rho GTPases, mitogen-activated protein kinase (MAPK), and phosphoinositide 3-kinase (PI3K) [16, 17]. TGF-*β*1 may act upon local activation by integrin *α*v*β*6, which can be expressed at high levels by epithelial cells during tissue repair. Specifically, integrin *α*v*β*6 cleaves the latency-associated peptide from the latent precursor of TGF-*β*1, which is otherwise sequestered in the ECM as inactive form. This mechanism of action is potentially relevant to promoting EMT changes, since, once locally activated, TGF-*β*1 exerts its effects only within the limits of the epithelial cells displaying this specific receptor [18]. Alternatively, EMT initiation and progression can be regulated by tyrosine kinase receptors (RTKs), involved in signal transduction of epidermal growth factor (EGF), fibroblast growth factor (FGF), insulin-like growth factor, hepatocyte growth factor (HGF), and platelet-derived growth factor (PDFG) [3]. RTKs stimulation has been widely linked to the activation of several regulatory molecules of EMT [19–23]. Notably, MAPK and PI3K pathways seem to play a major role in mediating RTKs-induced EMT [3]. Amongst morphogenetic signals, Wnt, Notch, and Hedgehog (Hh) signaling are well-established EMT inducers [24]. The ability of Wnt signals to trigger EMT relies on either the inhibition of the glycogen synthase kinase 3 (GSK3)-*β*, which prevents the destabilizing phosphorylation of Snail, or the nuclear translocation of *β*-catenin, whose gene targeting includes ZEB1 and Twist [25–27]. Notch and Hh signaling leading to the activation of EMT transcription factors (in particular the members of the Snail family) occurs through the activation of the Notch intracellular domain and the Gli family transcription factors, respectively [28–30].

The EMT program is finely regulated at a posttranscriptional level, by specific microRNAs (miRNA), including miR-1, miR-29b, miR-34, miR-200, and miR-203. These are small RNAs with about twenty nucleotides, regulating stability and translational activity of mRNAs. In particular, miRNAs act in double-negative feedback loops with several EMT transcription factors, wherein they repress the expression of each other, thus providing epithelial cells with an additional, finely tuned mechanism aimed at maintaining EMT. Furthermore, a direct effect of miRNAs has been shown on the expression of critical biomarkers, such as E-cadherin, vimentin, and fibronectin (e.g., miR-9, miR-138, and miR-17), as well as on several EMT-promoting ligands and their related signaling pathways (e.g., miR-200a for *β*-catenin, miR-204 for TGF-*β*R2, miR-15, miR-16 for FGF, miR-198 for HGF [31], and miR-181a for TGF-*β* [32]). Recent data indicate that miR-181a acts as a downstream effector of the TGF-*β* signaling in hepatocytes where it modulates the expression of a number of EMT-related genes, among which are E-cadherin and vimentin [32]. In cholangiocytes, miRNA-15a downregulates Cdc25a, a cell-cycle regulator with potent proliferative effects, a mechanism possibly involved in hepatic cystogenesis [33].

It is important to underline that phenotypic changes resulting in EMT can be triggered by disease mechanisms, such as inflammation, hypoxia, ECM remodeling, and autophagy. In fact, proinflammatory cytokines, such as tumor necrosis factor-*α* (TNF*α*) and interleukin-1*β*, and hypoxia can activate EMT master genes, acting through nuclear factor- (NF-) *κ*B and hypoxia inducible factor 1*α*, respectively [34].

The pathological remodeling of ECM also represents an additional mechanism of EMT progression [3]. In this regard, epithelial cells exposed to MMP-3 upregulate an alternative splice isoform of Rac1, which then enhances the expression of Snail by stimulating the production of reactive oxygen species [35]. Snail-induced EMT can be also triggered by type I collagen, which binds to *α*2*β*1 integrin causing an integrin linked kinase-mediated increase in nuclear NF-*κ*B activity [36].

Recent evidence suggest that also autophagy may behave as a critical regulator of EMT. Autophagy suppression by downmodulation of the autophagy-related gene 5 leads to the intracellular accumulation of the selective autophagy substrate p62, which then inhibits Twist1 protein degradation in both autophagosomes and proteasome, thereby decreasing E-cadherin expression and promoting cell motility [37, 38].

## 3. EMT in Liver Development

Acquisition of a mesenchymal phenotype endowed with migratory functions is a prerequisite of many morphogenetic processes. This concept is well established in renal biology, given the mesodermal origin of the tubular epithelium, while it is less defined in the liver, where, instead, the epithelial cells (hepatocytes and cholangiocytes) derive from the foregut endoderm, and the mesodermal contribution is restricted to the generation of the stromal cells, including hepatic stellate cells (HSC). Only scant evidence suggest an EMT role in liver development. Studies performed in the late 1990s showed that cultured hepatocytes from neonatal rat livers underwent EMT changes, represented by the loss of specific differentiation markers, gain of a migrating morphology, and replacement of typical hepatocyte cytokeratins by vimentin, a property further stimulated by EGF [39]. EMT features were then reported in both hepatocytes and progenitor cells isolated from rodent and human fetal livers [40, 41]. Studies in mice from Lemaigre's group addressed the hypothesis that EMT is critically involved in the early process by which endodermal cells that line the hepatic diverticulum migrate through the basement membrane to invade the septum transversum, where they give rise to the hepatoblasts in the liver bud [42]. This process is controlled by the hematopoietically expressed homeobox factor (Hex), which acts in concert with the transcription factor GATA-6; their downmodulation is essential for hepatoblast clustering after liver budding. Expression of the prospero-related homeobox 1 (Prox-1), likely stimulated by the T-box transcription factor 3 (Tbx3), is an additional mechanism critically involved in hepatoblast migration, which interplays with Hex (Hex-Prox-1 axis) [42]. Starting from this hypothesis, coexpression of K18 and *α*-SMA was found in most nonhematopoietic cells of human fetal livers at early gestational ages; furthermore, multipotent stem cells expressing EMT features along with the stem cell markers Oct4 and Nanog were isolated in the human liver bud [43]. Unlike the early ontogenetic steps, data supporting an involvement of EMT in the morphogenesis of intrahepatic bile duct epithelium are even less evident. Expression of the SRY-related HMG box transcription factor 9 (SOX9) is critical for differentiation to a biliary epithelial phenotype. SOX9 is early expressed in endodermal cells of the hepatic diverticulum, but it is then downregulated as these cells are invading the septum transversum. SOX9 is reexpressed in the hepatoblasts switching to the ductal plate cell phenotype, and it is then maintained by cholangiocytes during the progressive maturation of bile ducts. When SOX9 is defective, epithelial cells become hyperresponsive to TGF-*β* [44], thereby being susceptible to mesenchymal changes. Thus, it seems that cholangiocytes express an active program to suppress EMT.

## 4. In Chronic Cholangiopathies, Mesenchymal Markers Are Expressed by Ductular Reaction

Broadly speaking, whereas activation of an EMT program may play a physiological role in embryonic development [2], its actual impact in disease conditions evolving to scarring is quite controversial, particularly in the liver [45]. Cholangiopathies may provide important clues to clarify whether and how EMT may really contribute to liver fibrogenesis. Cholangiopathies are a heterogeneous group of genetic and acquired liver disorders primarily targeting the epithelial cell lining the bile ducts (cholangiocyte). Most cholangiopathies typically follow a chronic, progressive course, characterized by an excessive matrix deposition confined to the portal tract (portal fibrosis), starting from the closest peribiliary area, and ultimately leading to portal hypertension, often before the development of full-blown cirrhosis. In contrast with other chronic liver diseases, treatment of cholangiopathies is mainly symptomatic, reflecting the limited knowledge on their pathogenesis. Nowadays, liver transplantation remains the only curative opportunity, especially in children and young adults [46, 47].

Fibrogenesis is the main determinant of disease progression, as well as of the most severe clinical manifestations related to portal hypertension, in both chronic hepatocellular damage and cholangiopathies. Fibrogenesis is a consequence of the excessive and sustained activation of tissue repair mechanisms driven by the ductular reaction [1]. Ductular reaction is a dynamic, multicellular reparative system that includes mesenchymal and inflammatory cells accompanying the expansion of the epithelial cells lining the smallest ramifications of the biliary tree, in continuity with the canals of Hering, which is the niche where the hepatic progenitor cells (HPC) is thought to reside. Expansion of the HPC compartment is a compensatory mechanism of liver repair activated when proliferative ability of mature liver cells is compromised because of a severe liver damage [48]. HPC are small cells marked with the bipotential capability to differentiate towards both biliary and hepatocyte lineages [49]. In ductular reaction, HPC-derived epithelial cells are arranged into irregular, highly branched ductules devoid of lumen, generally extending into the liver parenchyma and along the margins of the portal tract. During this process, ductular cells express a “reactive” phenotype (ductular reactive cells, DRC) and acquire the ability to produce cytokines, chemokines, growth factors, and angiogenic factors and to express a rich repertoire of receptors typically displayed by ductal plate cells in the early stages of liver development [50]. Thanks to these phenotypic changes, DRC may establish intense paracrine communications with multiple stromal cell types, including myofibroblasts (MFs), inflammatory cells, and endothelial cells, which dictate the functional consequences of ductular reaction [1]. To set in motion this multicellular reparative complex, DRC acquire a high degree of cell plasticity. Therefore, DRC need first to reduce the strength of cell-cell and cell-matrix contacts and then to acquire motile functions, enabling them to move from the HPC niche towards the site of damage whereby, by interacting with other inflammatory and mesenchymal cell elements, they build up the ductular reaction. A mainstay of the migratory properties of DRC is their increased production of polysialic acid (PolySia) in the course of biliary damage. PolySia is a highly polar ECM structural component with a strong binding affinity to the neural cell adhesion molecule (NCAM), commonly expressed by DRC [51]. PolySia turns NCAM adhesive properties into antiadhesive due to the size of the multiple PolySia chains and their high hydrophilic content [52, 53]. This process is an essential step to promote plasticity and migration of NCAM^+^ cells in the generation of ductular reaction.

**Figure 1:**
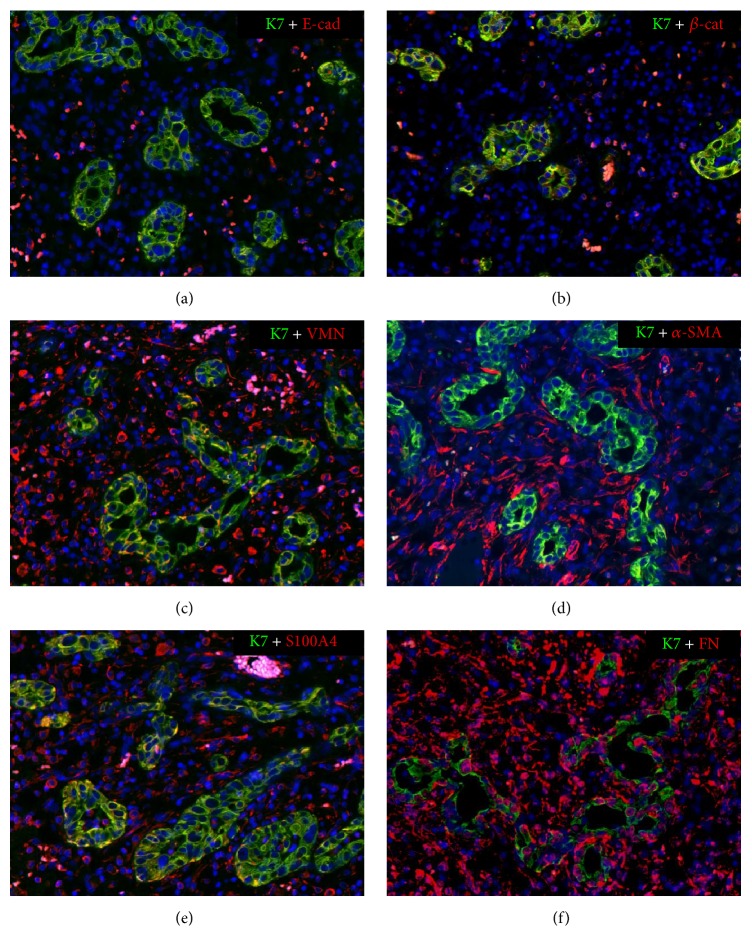
Partial expression of some mesenchymal features by ductular reactive cells. By dual immunofluorescence of a liver tissue section from a patient with ischemic cholangiopathy, with the cholangiocyte marker K7 (green), some mesenchymal features (red) are expressed by ductular reactive cells (coincident staining in yellow). They include downregulation of E-cadherin at the cell junctions of the epithelial layer (a) and upregulation in the cytoplasm of vimentin (c) and S100A4 (e) and in the basal side of fibronectin (f). In contrast, ductular reactive cells do not express typical markers of EMT, such as nuclear expression of *β*-catenin (b) and *α*-SMA (d) (M = 200x).

Although heavily involved in fibrogenesis, DRC lack the ability to actively secrete ECM components, such as type I or type IV collagen, and must cooperate with other effector cells by stimulating their profibrotic activities. Among them, DRC interactions with portal MFs are a crucial step in fibrogenesis [54]. Portal MFs are fibrogenic cells localized within the portal space, characterized by spindle-shape morphology, *α*-SMA expression, prominent motility, and contractility functions and strong capability to secrete ECM proteins, mostly type I collagen [55, 56]. They may originate from multiple cell sources, including HSCs and, to a lesser extent, portal fibroblasts and bone marrow-derived mesenchymal stem cells. These are recruited by paracrine signals (TGF-*β*, PDGF-B, vascular endothelial growth factor, angiopoietin-1, and sphingosine 1-phosphate) released in the site of damage by the DRC as well as by the other components of the ductular reaction, such as macrophages and inflammatory cells [1, 57, 58]. Whether the DRC themselves may be a further source of portal MFs via EMT as in the kidney [59, 60] and in the lung [61] has been hypothesized but never proven. However, several studies show that DRC express mesenchymal markers [62–64], as illustrated in Figure 1.

Initial studies showed that, in several cholangiopathies, cholangiocytes lining the small interlobular bile ducts and the reactive ductules lose some epithelial markers and acquire, in turn, several mesenchymal traits. Neoexpression of S100A4, vimentin, Snail, and MMP-2, associated with downregulation of E-cadherin and K19 in the bile ducts, were observed in histological samples of patients with primary biliary cirrhosis (PBC), primary sclerosing cholangitis [65], and biliary atresia (BA) [62, 66]. Reduced expression of E-cadherin and increased expression of vimentin and S100A4 were also reported in bile ducts of patients with intrahepatic lithiasis, where these phenotypic changes strongly correlated with the extension of biliary fibrosis [67]. Epithelial expression of mesenchymal markers has been reported also in animal models of biliary fibrosis induced by bile duct ligation (BDL). In the BDL rat, cholangiocytes upregulate the expression of S100A4 and downregulate the expression of the specific epithelial markers and the membrane channel aquaporin-1, together with the cytokeratins K7 and K19 [7]. Similarly, in the BDL mice, small clusters of cholangiocytes showed immunoreactivity for both *α*-SMA and heat-shock protein 47, a surrogate marker of type I collagen production, as well as migratory aspects into the periductal region [10]. However, it must be underlined that reliability of S100A4 as marker of EMT is limited by its concurrent expression by many inflammatory cells, including macrophages, often infiltrating the bile duct profile, thus posing a risk of misinterpreting histological sections.

Paracrine Hh signaling has been proposed by Omenetti and colleagues as a major driver of the EMT changes associated with biliary fibrosis in both rodents and humans [7]. In PBC, where EMT was originally proposed also as a mechanism contributing to ductopenia [68], Gli2, a transcription factor activated by Hh ligands, decorated the nuclei of ductular cells coexpressing both mesenchymal (S100A4, vimentin) and epithelial markers (K7) [7]. In BDL rats, relief of ductal obstruction reduced Hh pathway activity, an effect accompanied by reduction in EMT phenotype and biliary fibrosis [7]. Interestingly, mouse cholangiocytes cocultured with MFs, a rich source of soluble Hh ligands, acquired increased expression of several mesenchymal markers, including a migratory phenotype, while concomitantly repressing epithelial markers, and these effects were abolished by Hh antagonism [7]. Furthermore, EMT changes induced by BDL were exacerbated in transgenic mice harboring an overactivation of the Hh pathway caused by a defect in the Hh inhibitor Ptc [7]. The interplay between Hh activation and EMT was also reported in BA, a cholangiopathy featuring a pronounced ductular reaction associated with a rapid development of biliary fibrosis. In BA, a marked activation of the Hh signaling was associated with an excessive accumulation of ductular cells displaying an immature, mesenchymal-like phenotype responsive to Hh [69]. Hh ligands could be also secreted by hepatocytes both in human liver diseases and in mice models of liver damage. Hh ligands, Sonic and Indian Hh, both were overexpressed by hepatocytes in nonalcoholic steatohepatitis [70] and in chronic cholangiopathies such as primary biliary cirrhosis, respectively [71], in keeping with findings supporting a proapoptotic effect of Hh signaling [72]. Similarly, chronically liver injured mice by thioacetamide treatment showed an increased hepatocyte expression of Sonic and Indian Hh [73].

Noteworthy, Hh signaling may potentiate the pro-EMT effects of TGF-*β*1, by interacting with its downstream effectors at several levels (Smad3, Snail, and Twist), and, in turn, liver cell expression of Hh ligands can be stimulated by TGF-*β*1 [74]. The crosstalk between the two signaling pathways is particularly relevant in biliary fibrosis, since TGF-*β*1 is strongly upregulated in several cell types populating the ductular reaction, such as HSCs, endothelial cells, and Kupffer cells [1]. Given the effects on HPC and cholangiocytes, TGF-*β*1 seems to play a pivotal role in the generation of ductular reaction. Within the HPC niche, some cells display mesenchymal-like features, which are modulated by TGF-*β*1 [75]. At high doses, TGF-*β*1 is toxic to most epithelial cells, but, at low doses (1–10 ng/mL), it stimulates cultured cholangiocytes to acquire some mesenchymal markers, such as S100A4, vimentin, and *α*-SMA, to lose expression of K7, K19, and E-cadherin and to gain invading abilities through the basement membrane [10, 65, 66].

There is evidence suggesting that TGF-*β* may drive expression of mesenchymal markers in cholangiocytes also* in vivo* in specific disease settings. For example, cholangiocytes display some mesenchymal features in both rat [76] and mouse (personal data) models of congenital hepatic fibrosis (CHF), a genetic cholangiopathy caused by mutations of the ciliary protein fibrocystin. CHF is characterized by progressive peribiliary fibrosis, accompanied by biliary dysgenesia. In the CHF mouse model, fibrocystin-defective cholangiocytes possess increased migratory functions [9], upregulate integrin *α*v*β*6, and respond to TGF-*β*1, by producing collagen type I. This feature is not observed in cultured normal cholangiocytes and may contribute to matrix deposition in the adjacent peribiliary area, where fibrogenesis starts (personal data). These phenotypic changes appear to be dependent upon an activation of the *β*-catenin signaling caused by a noncanonical phosphorylation at Ser^675^. Ser^675^ phosphorylation prevents *β*-catenin from degradation, thereby allowing its nuclear translocation and, subsequently, its transcriptional activity. Activation of *β*-catenin as observed in fibrocystin-defective cholangiocytes is paradigmatic of the intracellular signaling perturbations induced by the loss of the tubular architecture and, consequently, of the cell polarity, which results in an increased secretion of cytokines, chemokines, and growth factors (personal data). This condition is likely reproduced by DRC, which, unlike normal cholangiocytes, are equipped with a number of ligand/receptor systems shared with the other cell elements involved in liver repair, mediating an extensive crosstalk ultimately leading to portal fibrogenesis [1].

**Figure 2:**
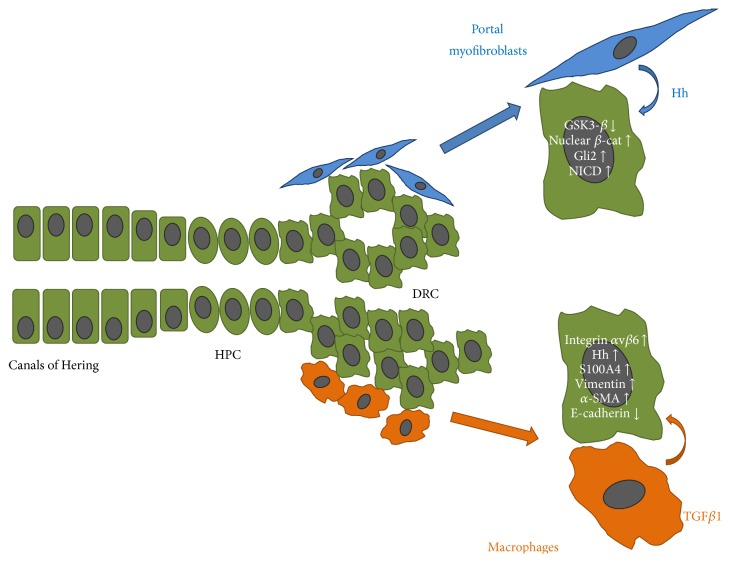
Epithelial-mesenchymal cell interactions promote ductular reaction. Crosstalk mechanisms with portal myofibroblasts and macrophages mediated by Hedgehog (Hh) and TGF-*β*1, respectively, are critical in generating ductular reactive cells (DRC) from activation of the hepatic progenitor cell (HPC) compartment, residing in the niche nearby the canals of Hering. Hh and TGF-*β*1 stimulate DRC to gain a range of mesenchymal changes typical of a “reactive” phenotype.

Even in the model of fibrocystin deficiency, mesenchymal-like changes are induced in cholangiocytes by TGF-*β*1 released in the peribiliary space by progressively infiltrating macrophages, whose recruitment is regulated by a range of chemokines (CXCL1, CXC10, and CXCL12) secreted by cholangiocytes in a *β*-catenin-dependent fashion. Importantly, this signaling perturbation is specific to fibrocystin deficiency, as it is not observed in polycystic liver diseases related to different ciliary protein defects, affecting polycystins [9]. However, even in this case, a full transdifferentiation of cholangiocytes into an activated mesenchymal phenotype (*α*-SMA neoexpression) was not found [76]. Crosstalk mechanisms driven by Hh ligands and TGF-*β*1 released in the inflammatory microenvironment, promoting mesenchymal changes in DRC, are outlined in Figure 2. Collectively, the pro-EMT body of evidence in cholangiopathies is summarized in Table 1.

## 5. Evidence against EMT in Biliary Fibrosis

Most studies supporting the occurrence of EMT in cholangiocytes are based essentially on a morphological approach, even when taking advantage of elegant* in vitro* methodologies and well-characterized animal models. These findings were not confirmed* in vivo* by lineage-tracing experiments. In these studies, mice harboring a Cre recombinase under a cholangiocyte- or oval cell-specific promoter were crossed with a reporter strain carrying the yellow fluorescence protein (YFP) reporter gene preceded a floxed Stop cassette, and the progeny was then subjected to a cholestatic, fibrogenetic liver injury, caused by BDL.

In the first study [11], using K19-CreERT × Rosa26-YFP mice, immunostaining revealed that after experimental liver injury, mesenchymal markers such as *α*-SMA, desmin (HSC biomarker), and S100A4 failed to colocalize in cholangiocytes tagged for K19 expression (i.e., YFP^+^ cells), although, within the portal tract, S100A4^+^ and K19^+^ cells localized in close proximity to each other. These data indicate that,* in vivo*, cholangiocytes do not activate an EMT program. These results were confirmed in MFs isolated from BDL livers, where no YFP^+^ cells could be detected. Similarly, in S100A4-green fluorescence protein (GFP) mice undergoing biliary damage, Pan-K^+^ cholangiocytes never overlapped with S100A4-GFP^+^ cells, thus confirming that cholangiocytes do not express S100A4. Even in this case, S100A4^+^ cells purified from cholestatic livers did not express panK, thus suggesting that a mesenchymal conversion of cholangiocytes, also transient, does not occur during liver injury. The discrepancy with Omenetti et al.'s paper [7] may be related to the different biliary marker (K7 versus K19) used for coexpression studies with S100A4 [11]. In fact, the cholangiocyte population is highly heterogeneous and a distinct subset of K19^+^/K7^−^ cholangiocytes in the terminal bile ductules, activated in specific disease conditions, has been identified [77]. On the contrary, a different subpopulation of K7^+^/K19^−^ cholangiocytes close to the HPC niche and mainly triggered by biliary damage has been also reported [78].

Therefore, to elude the technical trick related to the K19^+^ cell fate mapping, in a second study, Chu and colleagues used the alpha-fetoprotein (Alfp)-Cre × Rosa26-YFP mouse [8]. Taking this approach, the authors could track the cell fate not only of K19^+^ cholangiocytes, but also of HPC. Again, no evidence of YFP colocalization with the mesenchymal markers S100A4, vimentin, *α*-SMA, procollagen 1*α*2, or desmin was observed in liver tissue following BDL. Furthermore, no coexpression of the same markers by YFP^+^ cells could be observed after 3,5-diethoxycarbonyl-1,4-dihydrocollidine (DDC) diet, a model of biliary damage known to generate a robust HPC activation. Overall, these data support the finding that neither cholangiocytes nor their cell progeny may convert into fibrogenic MFs during experimental cholestatic liver injury. However, in contrast with the* in vivo* data, in the same study, cultured cholangiocytes isolated from the reporter mice and challenged with TGF-*β*1, alone or in combination with TNF-*α*, showed loss of cell-cell contacts along with a fibroblastoid cell reshaping, intracellular delocalization of E-cadherin, and increased expression of *α*-SMA. These apparent conflicting data clearly indicate that, under certain circumstances, cultured cholangiocytes may be committed to a complete EMT, unlike what happens in the* in vivo* condition, where the mesenchymal phenotype does not fully develop. This observation is in line with a recent study performed by our group to see if EMT contributes to the generation of the cancer-associated fibroblasts (CAF), usually accompanying the invasive growth of cholangiocarcinoma (CCA), a devastating malignancy originating from the biliary epithelium. A highly invasive human male CCA cell line (EGI-1) expressing an EMT phenotype, was xenografted by intraportal injection into a SCID male mouse, after transduction with lentiviral vectors encoding enhanced green fluorescence protein (EGFP). Liver tumors were analyzed by dual immunofluorescence for EGFP (serving as a CCA cell lineage marker) and *α*-SMA (CAF marker). Indeed, engrafted tumors were closely surrounded by abundant CAF, thus reproducing the native CCA characteristics. In this model, cancer cells that underwent a complete EMT would be expected to coexpress both markers. However, coincident labeling between EGFP and *α*-SMA was never observed in tissue samples from our xenograft models. Accordingly, FISH analysis further showed that the *α*-SMA^+^ cells expressed the murine rather than the human Y chromosome, which, instead, was normally expressed by infiltrating CCA cells [12].

Taken together, results of these fate-mapping studies are in accordance with a previous report showing the lack of EMT even in hepatocytes in a model of chronic hepatocellular damage [79] (Table 1). However, it must be underlined that experimental models of DDC and BDL are not fully consistent with the clinical phenotype of the human disease, most likely because of the rapid establishment of biliary fibrosis, which in chronic cholangiopathies takes instead several years to become clinically overt [45, 80, 81]. From this point of view, the CHF mouse model better reproduces the slow evolving tissue scarring seen in most human cholangiopathies.

## 6. Conclusions 

The controversy on the role of EMT in biliary fibrosis is substantially a matter of definitions [1]. The term EMT should be abandoned in cholangiocyte biology. Nevertheless, cholangiocytes may acquire, to a variable degree, some mesenchymal properties as part of a “reactive” phenotype, which develops without the concurrent loss of the native epithelial identity. As pointed out by Kriz and colleagues, the main caveat of several studies favoring the EMT hypothesis is the* a priori* assumption of EMT as an established fact, leading to misinterpretation of data that are compatible with but not evidence for EMT [82]. Therefore, the risk is to overlook the high complexity of a process whose relevance is recognized only in renal biology.

In the setting of a reactive phenotype (ductular reaction and biliary dysgenesis), ECM components are abnormally represented in close vicinity of bile ducts. This feature is typical of several chronic cholangiopathies and represents the mechanism leading to portal hypertension and its severe complications. Therefore, it is expected that hampering fibrosis progression would lead to a significant improvement of patient's survival. However, the availability of effective antifibrotic therapies is still remote [83], especially for primary cholangiopathies [47]. The identification of factors released in the inflammatory microenvironment and able to activate DRC as well as of signaling perturbations modulating mesenchymal changes may provide a wide range of putative novel targets (soluble factors, morphogens, transcription factors, and miRNA) whose therapeutic interference might halt the progression of biliary fibrosis, an issue worth being investigated by future studies.

## Figures and Tables

**Table 1:** Summary of evidence in favor of or against the existence of EMT in biliary diseases.

Model	Readouts	References
Pro-EMT		
Coculture of MFs and cholangiocytes	Cholangiocytes: ↑ S100A4, ↑ Fibronectin, ↑ N-cadherin, and increased motility	[7]
Cultured cholangiocytes from *α*-fetoprotein (Alfp)-Cre × Rosa26-YFP mice treated with TGF*β*, or TNF*α*	↑ *α*-SMA, loss of cell-cell contacts, cellular reshaping, and E-cadherin delocalization	[8]
Cultured cholangiocytes from* Pkhd1* ^*del4/del4*^ mouse	↑ motility due to *β*-catenin activation	[9]
BDL rat	Coexpression of S100A4 and vimentin with K7	[7]
BDL rat	DRC (immunohistochemistry): ↑ S100A4, ↑ heat-shock protein 47, ↑ *α*-SMA, ↓ K7, ↓ K19, and ↓ Aquaporin-1	[10]

Against-EMT		
K19-CreERT × Rosa26-YFP mice, BDL	No coexpression of K19 YFP with *α*-SMA, Desmin, or S100A4	[11]
S100A4-CreERT × Rosa26-YFP mice, BDL	No coexpression of S100A4-GFP with Pan-K cells	[11]
*α*-fetoprotein (Alfp)-Cre × Rosa26-YFP mice, BDL	No coexpression of YFP with S100A4, vimentin, *α*-SMA, procollagen 1*α*2, or desmin	[8]
*α*-fetoprotein (Alfp)-Cre × Rosa26-YFP mice, DDC	No coexpression of YFP with S100A4, vimentin, *α*-SMA, procollagen 1*α*2, or desmin	[8]
Human EGI-1-EGFP xenograft in SCID mice	No K19/*α*-SMA coexpression; no expression of Y human chromosome on *α*-SMA^+^ cells	[12]
